# Forced mobilization accelerates pathogenesis: characterization of a preclinical surgical model of osteoarthritis

**DOI:** 10.1186/ar2120

**Published:** 2007-02-06

**Authors:** C Thomas G Appleton, David D McErlain, Vasek Pitelka, Neil Schwartz, Suzanne M Bernier, James L Henry, David W Holdsworth, Frank Beier

**Affiliations:** 1 CIHR Group in Skeletal Development & Remodeling, Schulich School of Medicine & Dentistry, The University of Western Ontario, London, Ontario, N6A 5C1, Canada; 2 Department of Physiology & Pharmacology, Schulich School of Medicine & Dentistry, The University of Western Ontario, London, Ontario, N6A 5C1, Canada; 3 Imaging Research Laboratories, Robarts Research Institute, London, Ontario, N6A 5C1, Canada; 4 Department of Medical Biophysics, Schulich School of Medicine & Dentistry, The University of Western Ontario, London, Ontario, N6A 5C1, Canada; 5 Micheal G DeGroote Institute for Pain Research & Care, McMaster University, Hamilton, Ontario, L8S 4L8, Canada; 6 Department of Anatomy & Cell Biology, Schulich School of Medicine & Dentistry, The University of Western Ontario, London, Ontario, N6A 5C1, Canada; 7 Department of Diagnostic Radiology & Nuclear Medicine, Schulich School of Medicine & Dentistry, The University of Western Ontario, London, Ontario, N6A 5C1, Canada

## Abstract

Preclinical osteoarthritis (OA) models are often employed in studies investigating disease-modifying OA drugs (DMOADs). In this study we present a comprehensive, longitudinal evaluation of OA pathogenesis in a rat model of OA, including histologic and biochemical analyses of articular cartilage degradation and assessment of subchondral bone sclerosis. Male Sprague-Dawley rats underwent joint destabilization surgery by anterior cruciate ligament transection and partial medial meniscectomy. The contralateral joint was evaluated as a secondary treatment, and sham surgery was performed in a separate group of animals (controls). Furthermore, the effects of walking on a rotating cylinder (to force mobilization of the joint) on OA pathogenesis were assessed. Destabilization-induced OA was investigated at several time points up to 20 weeks after surgery using Osteoarthritis Research Society International histopathology scores, *in vivo *micro-computed tomography (CT) volumetric bone mineral density analysis, and biochemical analysis of type II collagen breakdown using the CTX II biomarker. Expression of hypertrophic chondrocyte markers was also assessed in articular cartilage. Cartilage degradation, subchondral changes, and subchondral bone loss were observed as early as 2 weeks after surgery, with considerable correlation to that seen in human OA. We found excellent correlation between histologic changes and micro-CT analysis of underlying bone, which reflected properties of human OA, and identified additional molecular changes that enhance our understanding of OA pathogenesis. Interestingly, forced mobilization exercise accelerated OA progression. Minor OA activity was also observed in the contralateral joint, including proteoglycan loss. Finally, we observed increased chondrocyte hypertrophy during pathogenesis. We conclude that forced mobilization accelerates OA damage in the destabilized joint. This surgical model of OA with forced mobilization is suitable for longitudinal preclinical studies, and it is well adapted for investigation of both early and late stages of OA. The time course of OA progression can be modulated through the use of forced mobilization.

## Introduction

Osteoarthritis (OA) is a complex degenerative disease [1-3] that causes structural changes to articular cartilage and subchondral bone of synovial joints [4-7]. An understanding of OA etiopathology, however, has proven to be elusive [2]. Coupled with the fact that OA affects nearly 70% of all people at some point in their lives, OA has major economic and social impacts on patients and health care systems [8-10]. Consequently, there is a pressing need to develop disease-modifying OA drugs (DMOADs).

Before a DMOAD can reach clinical trials, it must first be successful in preclinical trials. This requires animal models of OA in which specific aspects of drug efficacy in articular cartilage, subchondral bone and other affected tissues may be examined, as may potential side effects in other organs [11]. Large animals such as dogs or sheep are sometimes preferred for these purposes because they provide sufficient amounts of tissue for analysis [12]. However, large animal studies incur high costs (for instance, housing), which make them impractical for large-scale screens of multiple compounds. In contrast, small animals (such as rodents) are more cost-effective than large ones, and they are well suited to longitudinal preclinical OA studies. Among these, rats and mice are particularly promising because of advanced annotation of their genomes and the remarkable genetic, anatomic, and physiologic similarities between humans and rodents [13].

Rodent models of OA were first developed in the late 1970s in mice and rats [14-17]. Initially, experiments employed models in which OA was induced in the temporomandibular joint [18-20], but subsequently these models were developed to involve other synovial joints, including the knee [14]. Either a chemical method (intra-articular injection of, for instance, papain [21] or sodium iodoacetate [22]) or a surgical method (structural alteration to the tendons, muscle, or ligaments [23-25]) was used. A review by Shwartz [26], published in 1987, summarizes these early developments. Other models developed since then rely on genetic predisposition or engineering to stimulate OA pathology. However, a long time may be required for OA to develop in genetic models, and there is often considerable variability between animals (for example, in the temporal dynamics of OA progression). Disease progression in surgical models is faster and more consistent. Moreover, these models reflect post-traumatic (secondary) OA, because they rely on changes in weight bearing and unnatural joint articulation for OA etiopathology [27,28].

It is advantageous to develop surgical models in rats or mice because genetic studies are possible in these animals [29-31]. Rat models are of interest because their larger size (compared with mice) provides more tissue for biochemical and gene expression analysis, and permits cross-disciplinary studies (for example, genomics, cell biology, electrophysiology, and *in vivo *small animal imaging) [32]. Models developed in the rat include anterior cruciate ligament transection (ACL-T) [33-35] and partial meniscectomy (PM) [36,37], or a combination of both [38]. Only a few groups have characterized aspects of rat OA model pathology. For example, Hayami and coworkers [31] recently assessed the combination of ACL-T with PM. However, comprehensive longitudinal characterization of OA progression, from early to late stages (evaluating articular and subchondral lesions, volumetric bone mineral density [vBMD], and biochemical markers of cartilage breakdown), has not been performed. Furthermore, although specific exercise protocols [35,39] are believed to alter OA pathogenesis, longitudinal evaluation of forced mobilization (FM) has never been investigated.

Recent advances in *in vivo *small animal imaging have allowed us to quantify changes in subchondral bone over time [32] (McErlain and coworkers, unpublished data). We have shown that this model develops OA-like changes in subchondral bone microarchitecture. However, detailed characterization of disease progression at multiple levels is required before this model may be utilized in preclinical DMOAD studies. We also hypothesized that FM in this model would cause late-stage OA to develop more quickly, thus accelerating studies targeting late-stage OA. Here, we report a comprehensive evaluation of our preclinical surgical model of OA and the effects of FM on pathogenesis. We used quantitative methods to assess both cartilage [40-42] and subchondral bone [34,43-45] pathology in early-stage [31,46] and late-stage [47,48] OA. We anticipate that this study will facilitate preclinical trials evaluating DMOAD efficacy and will be of benefit to those evaluating the outcomes of subsequent clinical studies.

## Materials and methods

### Surgical rat model of osteoarthritis

Surgery was performed only on the right knee of weight-matched male Sprague-Dawley rats (Charles River Laboratories, St. Constant, Quebec, Canada) in the study. Animals were allowed to reach a body weight of 300 to 325 g before surgery. Anesthetic (50% ketamine [100 mg/ml], 25% xylazine [20 mg/ml], 10% acepromazine [10 mg/ml], and 15% saline [0.9% solution]) and Trisbrissen antibiotic (Schering Canada, Inc., Pointe Claire, Quebec, Canada) were both administered at a dose of 100 μl per 100 g body weight.

The surgical protocol was carried out as follows. Ninety-six animals were equally divided into one of two treatment groups (Table 1): sham (control) and OA group. The OA treatment group underwent open surgery involving anterior cruciate ligament transection (ACL-T) and partial medial meniscectomy (PM) via an incision on the medial aspect of the joint capsule, anterior to the medial collateral ligament. Following surgery, the incision was closed in two layers. The joint capsule was sutured independently from peripheral tissues using dissolvable 5-0 Vicryl sutures, and the skin closed by interrupted sutures using 5-0 braided silk (Ethicon, Johnson & Johnson Medical Products, Markham, Ontario, Canada). This treatment was used to induce OA pathogenesis, and the operated joint hereafter is referred to as the 'ipsilateral' treatment. Conversely, the left (nonoperated) knee joint is referred to as the 'contralateral' treatment. The second group of rats underwent a sham operation in which a similar incision in the joint capsule was made but ACL-T and PM were not performed. Only the right knees of sham animals were used as controls for disease progression.

After surgery, all animals were administered antibiotics and analgesics in accordance with standard operating protocols established by the Animal Care and Use Committee at the University of Western Ontario. Four animals were used per time point in each treatment group. These animals first underwent micro-computed tomography (CT) analysis and were then killed at 2, 4, 8, 12, 16, or 20 weeks after surgery. Knee joint tissues were processed for histologic evaluation. Preliminary micro-CT scans and histology were done on a group of 300 to 325 g animals before surgery. It was found that the 2-week sham vBMD and histology were similar to those at the presurgical time point (data not shown). Animal experiments were approved by the Animal Care and Use Committee at the University of Western Ontario. All animals used in the study remained healthy throughout the experiments. None of the animals exhibited any overt change in feeding behavior or activity as a result of their surgery. Weight gain over the 5-month time course was similar in both groups (*P *= 0.058). The mean body weights (± standard error) of the nonmobilized group and FM group were 618.8 ± 13.54 g and 655.3 ± 7.825 g, respectively.

### Forced mobilization protocol

**Figure 1:**
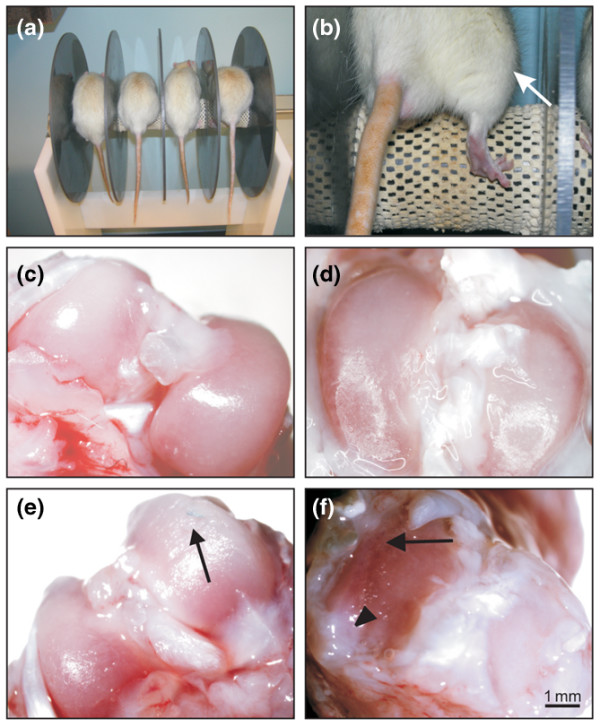
Forced mobilization apparatus and macroscopic analysis of joint degradation. **(a) **Following sham (control) or OA surgery, FM animals underwent forced mobilization. Animals walked on a rotating cylinder for 30 min, three times per week. **(b) **FM forces the maximal extension and flexion of the knee joint (white arrow). To assess macroscopic changes to the articular surface, knee joints were dissected 4 weeks after surgery and photographed. Representative images from sham **(c) **tibias and **(d) **femurs, and ipsilateral **(e) **tibias and **(f) **femurs are shown. Surface abrasions (black arrow) and fibrotic tissue (arrow head) were observed in ipsilateral surfaces, compared with the smooth, glassy appearance in shams. Scale bar applies to panels c-f. FM, forced mobilization; OA, osteoarthritis.

Twenty-four of the animals from both treatment groups (48 animals) underwent FM, beginning 5 days before surgery to train the animals. The remaining 24 sham and 24 OA animals did not undergo FM and are referred to as 'nonmobilized' (NM). Table 1 summarizes the surgical and mobilization treatments. Forced mobilization was used to force weight bearing, flexion, and extension of the knee joint for a given period of time. For FM experiments, a rotating cylinder apparatus [49,50] was constructed consisting of a motor-driven rotating cylinder (8 cm diameter) covered with cotton mesh (for grip), which was divided into lanes and suspended 1 m above the ground (Figure 1a). The cylinder rotated toward the animals at a rate of 4 rpm. This device forced the animals to flex and extend both hind-limb knee joints maximally as they walked on the cylinder (Figure 1b). Each animal completed a 30 min session of FM on Mondays, Wednesdays, and Fridays each week.

### Micro-CT analysis

#### Micro-CT scanning

*In vivo *micro-CT using a General Electric Health Care eXplore Locus scanner (GE Health Care Life Sciences, Baie d'Urfe, QC, Canada) was carried out at each time point on every animal, before they were killed and tissues underwent histologic processing. Animals were anesthetized as described above and placed in the scanner in a supine position. An epoxy-based cylinder (1 mm diameter) attached to the limb to be scanned was used for calibration (SB3; Gamex RMI, Middleton, WI, USA). The X-ray tube has a tungsten target with a nominal spot size of 50 μm and 1.8 mm A1-equivalent filtration. We obtained X-ray acquisition images at 1° increments over 210°, from a summation of five X-ray projections (400 ms/exposure) with 80 kVp and 450 μA exposure parameters. Each acquired image was subsequently corrected using a bright-field and dark-field image. Data reconstruction with a modified Feldkamp conebeam algorithm [51] resulted in three-dimensional micro-CT images with an isotropic voxel spacing of 46 μm × 46 μm × 46 μm. Total micro-CT volume was calibrated in Hounsfield units, and the total scan time for both hind limbs was approximately 17 min.

#### Data analysis with MicroView software

General Electric Health Care MicroView software was used to analyze the multiplanar reformatted images in axial, coronal, and sagittal planes. Each scan was monitored quantitatively for changes in vBMD and qualitatively for the presence of osteophytes, subchondral cysts, and heterotopic ossification. Using our previously developed spatial sampling method (McErlain and coworkers, unpublished data), we divided the joint into two medial compartments: the medial femoral compartment (MFC) and medial tibial plateau (MTP). The vBMD for each compartment was calculated as follows. Each compartment was analyzed using anterior, central, and posterior regions of interest (ROIs) allowing averaging of three sampling areas per joint compartment [52]. A primary 'Y' axis was assigned based on the anterior and posterior margins of each compartment, and a secondary 'X' axis was assigned based on the medial and lateral margins of each compartment. A 2 × 4 grid divided the primary 'Y' axis into four quarters and the secondary 'X' axis into halves. The central ROI was assigned to the intersection between Y2 and X2. The anterior and posterior ROIs were adjusted to accommodate the natural curvature of the bones by ensuring that the medial and lateral borders of each ROI did not extend beyond the bone-tissue interface. The patellofemoral and tibial tuberosity regions were avoided. The Z position (depth of the ROI) was set as the minimum distance between the subchondral and epiphyseal plates and varied between tibia and femur. ROIs with a diameter of 0.75 mm were sampled at a depth of 0.85 mm in each tibial compartment and a depth of 1.5 mm in each femoral compartment.

### Processing of histologic samples

Four animals from both treatment groups of NM and FM animals (16 animals) were anesthetized as above before transcardial perfusion first with saline and then 4% with paraformaldehyde at each time point. The hind limbs were dissected *ex vivo *above and below the knee joint and placed in a 0.4 M EDTA, 0.3 N NaOH, and 1.5% glycerol (pH 7.3) solution, which was changed every 3 days, for 4 to 5 weeks of decalcification (end-point determined by physical assessment). All processing and sectioning of the knee joints was carried out at the Robarts Research Institute Molecular Pathology Laboratory (London, Ontario, Canada). Each joint was embedded in paraffin wax and sectioned in the sagittal plane starting from the medial margin of the joint. Serial sections with a thickness of 6 μm were taken, beginning with the 30th section from the medial joint edge. Every fifth section from this starting point was kept until 40 slides were obtained. Of these, every fifth slide was selected for staining with 0.1% safranin-O, 0.02% fast green, and Harris' haematoxylin counterstaining. The site of the partial meniscectomy (the medial joint compartment) was selected for analysis in these studies. A total of eight stained sections per sample, spanning 1.2 mm of each medial compartment, was used for histologic scoring.

### Scoring of histological samples

The Osteoarthritis Research Society International (OARSI) scoring method [42] was used to assess and compare the progression of OA in all samples. The grade and stage of both the tibia and femur were assessed independently in at least five stained slides from each sample by a blinded observer. OA score was calculated by multiplication of the grade and stage values for each slide. A minimum score of 0 indicates no OA activity and a maximum score of 24 indicates the highest degree of OA activity in more than 50% of the section, where OA activity is defined by the presence of OA-like features including surface discontinuity, loss of proteoglycans, among other features. The score for each medial compartment joint surface was assigned by determining the average score of all slides assessed from that sample. Four replicates per time point, per treatment, per joint surface (tibial and femoral joint surfaces were assessed independently) were used to calculate the overall score means.

### Assessment of collagen breakdown by CTX II urinalysis

An independent group of animals was used to assess type II collagen breakdown by quantifying CTX II fragments in urine [53]. Twenty animals underwent either sham or ACL-T/PMM (OA) surgery as described above and five animals from each surgical treatment were randomly assigned to either NM or FM groups. Morning spot urine samples were obtained from sham NM, sham FM, OA NM, and OA FM individuals at presurgical, 2, 4, 8, 12, and 16 week time points for repeated measurement of urine CTX II. Urine was sampled on FM treatment days, just before FM treatment. A Urine Pre-Clinical CartiLaps^® ^enzyme-linked immunosorbent assay (Nordic Biosciences, Herlev, Denmark) was used to measure the levels of CTX II in urine over time, in accordance with the manufacturer's protocol. Standards and samples were assayed in duplicate on 96-well plates. Absorbance was measured at 450 nm, with 600 nm as a reference wavelength, to quantify CTX II in the samples. Nonlinear regression analysis of log-transformed concentration values was used to construct a standard curve with standard absorbance readings. Averaged absorbance readings were then used to interpolate CTX II levels in urine samples using the standard curve. To correct for variations in urine concentration between animals, CTX II concentration was normalized to creatinine in each sample. Creatinine concentration was determined using a Creatinine Assay Kit (Oxford Biomedical Research, Inc., Oxford, MI, USA) based on the Jaffe reaction [54,55], in accordance with the manufacturer's protocol. Standard and sample creatinine concentrations were determined in 96-well plates from 450 nm absorbance readings. Averaged absorbance readings were used to interpolate creatinine concentration in each urine sample from a standard curve. Corrected CTX II values are expressed in micrograms of CTX II per millimoles of creatinine.

### Immunohistochemistry

Additional 6 μm sections from the medial compartment of each joint were used for immunohistochemical analyses in FM joints up to 20 weeks. Primary antibodies against matrix metalloprotein (MMP)-13 (Cedarlane Labs, Hornby, Ontario, Canada), alkaline phosphatase (Abcam, Cambride, MA, USA), or type X collagen (Sigma, Oakville, Ontario, Canada), followed by secondary antibodies conjugated to horseradish peroxidase, were used to detect the expression of each protein within the articular knee cartilage and growth plates (positive control) of ipsilateral, contralateral, and sham knees at each time point after surgery. Colourimetric detection with DAB substrate (Dako USA, Carpinteria, CA, USA) was carried out for equal time periods in sections probed with the same antibody, and Harris' hematoxylin was used as a nuclear counterstain. Detection of each protein was carried out on sections from at least three different animals per treatment group. Slides incubated without primary antibody were used as negative controls.

### Statistical analyses

The statistical analysis program Graph Pad Prism 4.0 (Graph Pad Software Inc., San Diego, CA, USA) was used for all statistical tests. Statistical tests on OARSI histologic grading and staging scores, vBMD values, and CTX II level datasets were performed with two-way analyses of variance to determine whether the effect of surgical group or time point was significant. In addition, one-way analysis of variance using Tukey's *post hoc *tests was used to compare means between all surgical groups at each time point and between all time points for each group. All values are expressed as the mean ± standard error. *P *< 0.05 was considered statistically significant.

## Results

### Longitudinal study of histological changes in articular joint degradation

We examined operated knee joints macroscopically, 4 weeks after surgery. A healthy articular surface was observed in all sham animals, whereas in model animals ipsilateral joint surfaces were abraded and contained fibrotic tissue (Figure 1c–f). Contralateral surfaces appeared similar to those in sham knee joints (not shown). This confirmed that ACL-T and PM surgery induced OA-like degradation of the articular surface.

**Figure 2:**
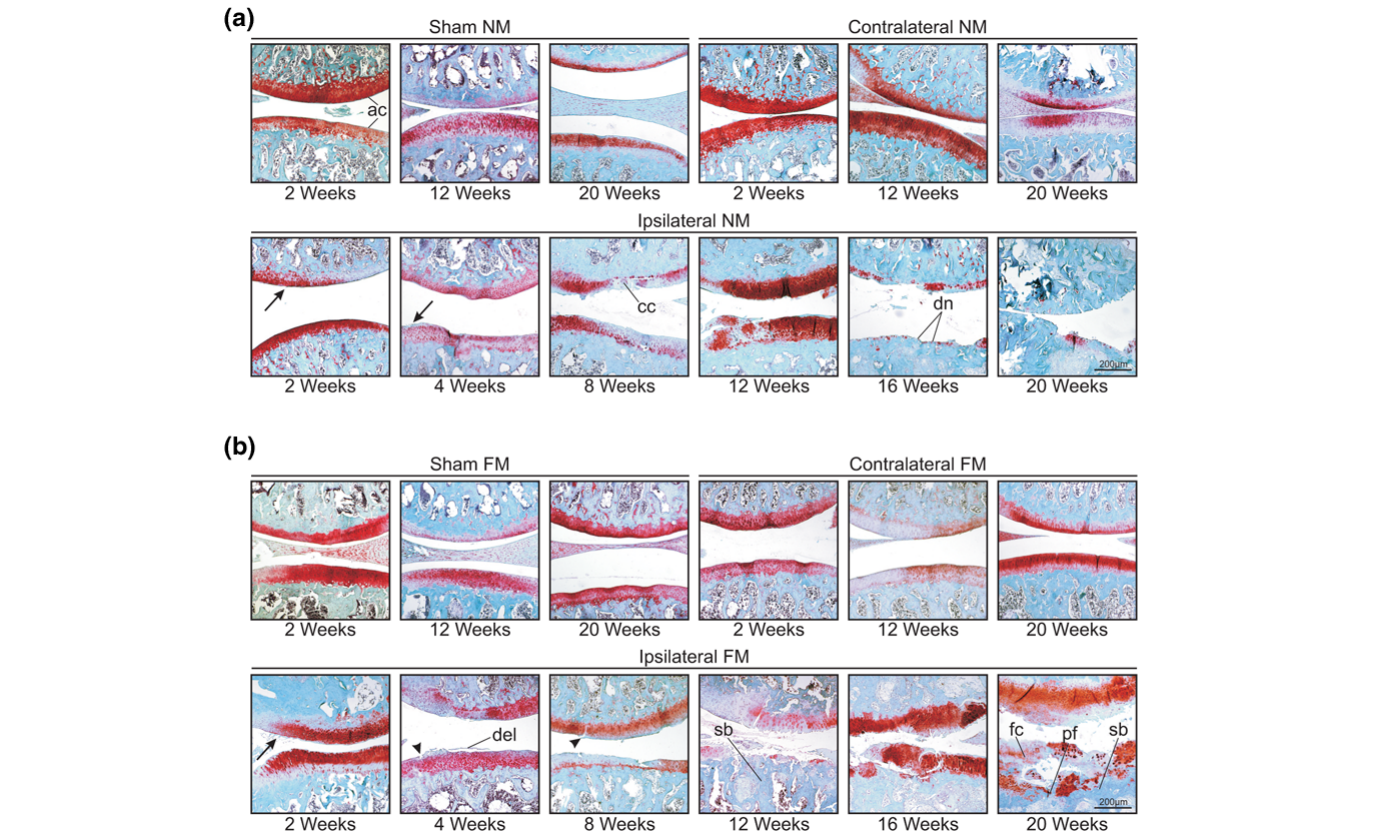
Histologic analysis over time reveals patterns of articular degradation. Sagittal sections from sham (control), and contralateral and ipsilateral OA treatments, in **(a) **nonmobilized (NM) and **(b) **forced mobilization (FM) groups of animals were analyzed over a 20-week time course. Sections were stained with safranin-O (red stain) for articular cartilage matrix proteoglycans, fast green (green stain) for bone and fibrous tissue, and hematoxylin for nuclei (blue). In the upper row of each panel, representative images of sham and contralateral joints are shown at 2, 12, and 20 weeks after surgery. The lower row shows representative sections of ipsilateral joints at all time points assessed. Each image is presented with the femoral joint surface in the upper portion. Examples of morphologically normal articular surface (ac), surface discontinuity (arrow), vertical fissures (arrow head), delamination (del), chondrocyte clusters (cc), denudation (dn), sclerotic bone (sb), fibrocartilage-like tissue (fc), and subchondral plate failure (pf) are indicated. All images are shown at the same magnification, indicated by the scale bars.

**Figure 3:**
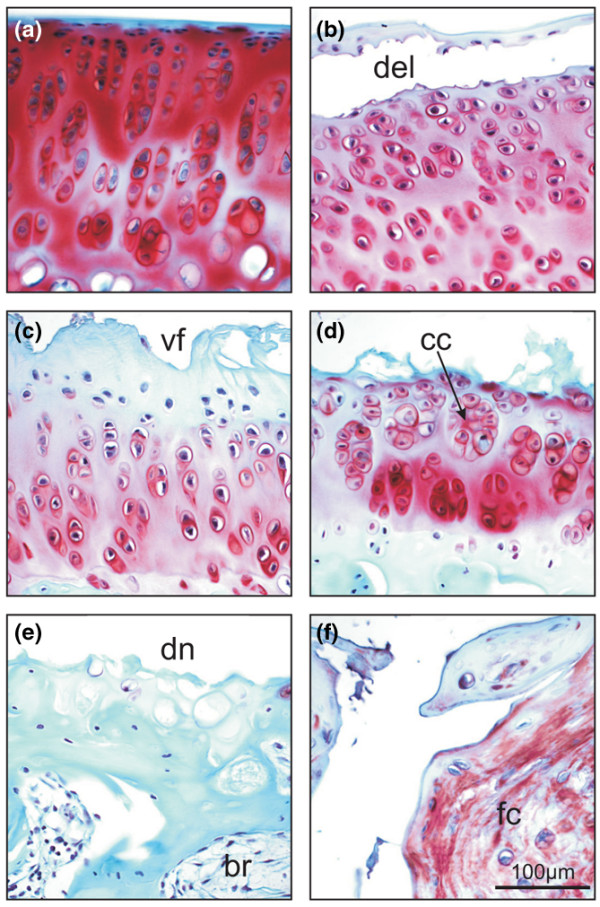
High magnification images of sagittal sections of articular cartilage, stained with safranin-O and fast-green, reveal detailed cartilage histology. **(a) **Healthy-appearing sham cartilage has intact superficial, mid, and deep zones (from top to bottom of image) that stain deeply with safranin-O (red) for glycosaminoglycans. The chondrocytes are arranged in columns. **(b) **Two week FM ipsilateral cartilage demonstrates delamination (del) of the superficial zone. **(c) **Four week FM ipsilateral cartilage shows the development of vertical fissures (vf) into the mid-zone, and loss of glycosaminoglycans (pale green stain in mid-zone is red in panel a). **(d) **Matrix erosion of the superficial and mid-zones is evident by 8 weeks in FM ipsilateral cartilage, as well as the formation of chondrocyte clusters (cc). **(e) **By 16 weeks, NM ipsilateral cartilage shows almost complete denudation (dn) of the articular cartilage, and evidence of bone repair appears beneath the subchondral plate (br). **(f) **Fibrocartilage-like tissue (fc) is evident in the articular cartilage of 20-week FM ipsilateral joints, which is indicative of abnormal repair processes. All images are shown at the same magnification, indicated by the scale bar. FM, forced mobilization; NM, nonmobilized.

**Figure 4:**
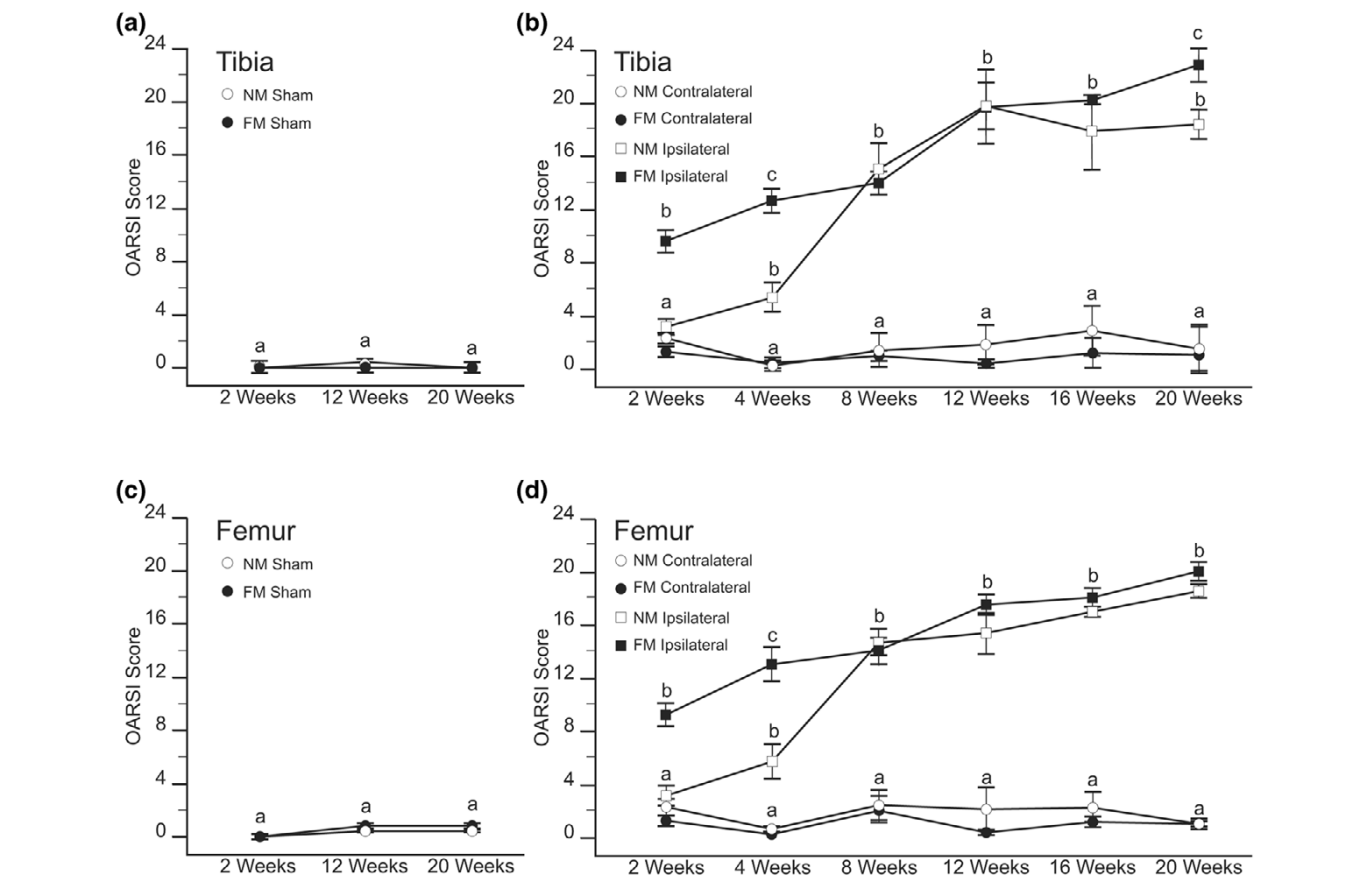
OARSI histopathology grading and staging scores. OARSI histopathology grading and staging scores were determined in sham (control), and contralateral and ipsilateral treatments of both NM and FM groups of animals over 20 weeks. Tibial joint surfaces from **(a) **sham and **(b) **contralateral and ipsilateral treatments were assessed independently of femoral **(c) **sham and **(d) **contralateral and ipsilateral joint surfaces. Mean OARSI scores ± standard error are shown. Significantly higher scores were observed in NM contralateral femurs than NM sham femurs at 2 and 12 weeks (statistics not shown). Both ipsilateral surfaces had significantly higher OARSI scores than shams at all time points, except NM ipsilateral surfaces at 2 weeks (statistics not shown). Statistical analysis is done for each individual time point to indicate significantly different means among each of the four contralateral and ipsilateral treatments. Similar means at each time point are indicated by the same letter (a, b, and c), whereas significantly different means at each time point are indicated by different letters (*P *< 0.05; *n *= 4). FM, forced mobilization; NM, nonmobilized; OARSI, Osteoarthritis Research Society International.

We then carried out histologic analysis of sham, contralateral, and ipsilateral joints at several time points up to 20 weeks after surgery (Figure 2). Healthy articular cartilage has a smooth, uninterrupted surface and an even distribution of chondrocytes often arranged in columns [56]. The extracellular matrix has a rich distribution of proteoglycans and glycosaminoglycans [57]. Sham joints exhibited a healthy appearance throughout the duration of the study (Figures 2 and 3a). OARSI histopathology scoring confirmed these observations. Near-zero OARSI scores indicated that OA activity did not occur at any point up to 20 weeks in either joint surface (tibial or femoral) of NM and FM sham animals (Figure 4a,c).

A small degree of degradation (such as proteoglycan loss) was detected in FM and NM contralateral joint surfaces at 12 and 20 weeks, respectively (Figure 2). These changes, however, did not worsen over time, and inter-animal variations were minor. The OARSI scores of contralateral joints confirmed no progression of degradation in either joint surface, with or without FM. For example, there was a significantly higher NM contralateral femur score, compared with NM sham femurs, at 2 and 12 weeks but not 20 weeks (statistics not shown). Finally, no significant differences between NM and FM contralateral scores were observed for either tibial or femoral surfaces (Figure 4b,d). These results indicate that the contralateral joint develops minor but nonadvancing morphologic OA characteristics up to 5 months after surgery.

Degradation in ipsilateral joints, conversely, was severe for both joint surfaces. NM animals exhibited surface discontinuity of both joint surfaces by 2 weeks, which extended across less than half of the articular surface (Figure 2a). Similar degradation was also observed at the 4-week time point in NM animals (Figure 2a). In FM animals, however, early degradation was greater. Surface discontinuity was present over most of the articular surface at 2 weeks, including shallow vertical fissures through the cartilage superficial zone at many points across the surface (Figure 2b) and delamination of the superficial zone (for example, see Figure 3b) was restricted to small focal regions. An even greater extent of degradation was seen in FM animals than in NM animals after 4 weeks (Figure 2). For example, there was an increase in vertical fissure formation and depth into the mid-zone (for eample, see Figure 3c), and chondrocyte clusters appeared in the mid-zone (for example, see Figure 3d). Similar changes did not occur in NM joint surfaces until 8 weeks (Figure 2a).

Proteoglycan loss occurred early in both NM and FM ipsilateral joint cartilage (Figure 2). Interestingly, loss of proteoglycans in a particular region correlated with the presence of more advanced lesions in that region than peripherally. Over the whole time course, however, proteoglycan loss was not progressive. For example, proteoglycan staining was consistently stronger at 16-week to 20-week time points in FM ipsilateral joints than at early to moderate stages (8 to 12 weeks), particularly in regions where repair tissue was present (Figure 2b).

Our qualitative observations were reflected in ipsilateral joint OARSI scores (Figure 4b,d). First, significantly higher scores were observed in both NM and FM tibial and femoral ipsilateral joint surfaces at all time points, with the exception of NM ipsilateral surfaces at 2 weeks (similar to NM contralateral score), as compared with all contralateral and sham scores (statistics not shown). Next, significantly higher scores in both FM ipsilateral joint surfaces were observed at 2 and 4 weeks compared with NM animals (Figure 4b,d). OA activity in NM and FM animals converged by 8 weeks and continued to increase at similar rates in both NM and FM groups up to 16 weeks. Between 8 and 16 weeks, surface discontinuity, vertical fissures (Figure 3c), proteoglycan loss (loss of staining; Figure 3c), and chondrocyte clusters (Figure 3d) were seen in both joint surfaces (Figure 2). However, by 20 weeks in FM joint surfaces there was far greater deformation of the cartilage surface (Figure 2b) than in the NM group (Figure 2a), which mainly exhibited denudation (Figure 3e). This included evidence of subchondral bone repair (Figure 3e), sclerotic subchondral bone (Figure 2b), fibrocartilage-like tissue within the cartilage surface (Figure 3f), and osteophyte formation at the joint margins. Significantly higher OARSI scores (*P *= 0.029) were observed in FM ipsilateral tibial surfaces (22.375 ± 0.718) than in NM ipsilateral tibias (18.425 ± 0.394) at 20 weeks (Figure 4b). Taken together, these results indicate that destabilization surgery induces OA activity as early as 2 weeks after surgery, and is accelerated by FM during early OA development. Later, FM results in quantifiably greater joint deformation, particularly in tibial joint surfaces.

### Longitudinal analysis of subchondral bone

**Figure 5:**
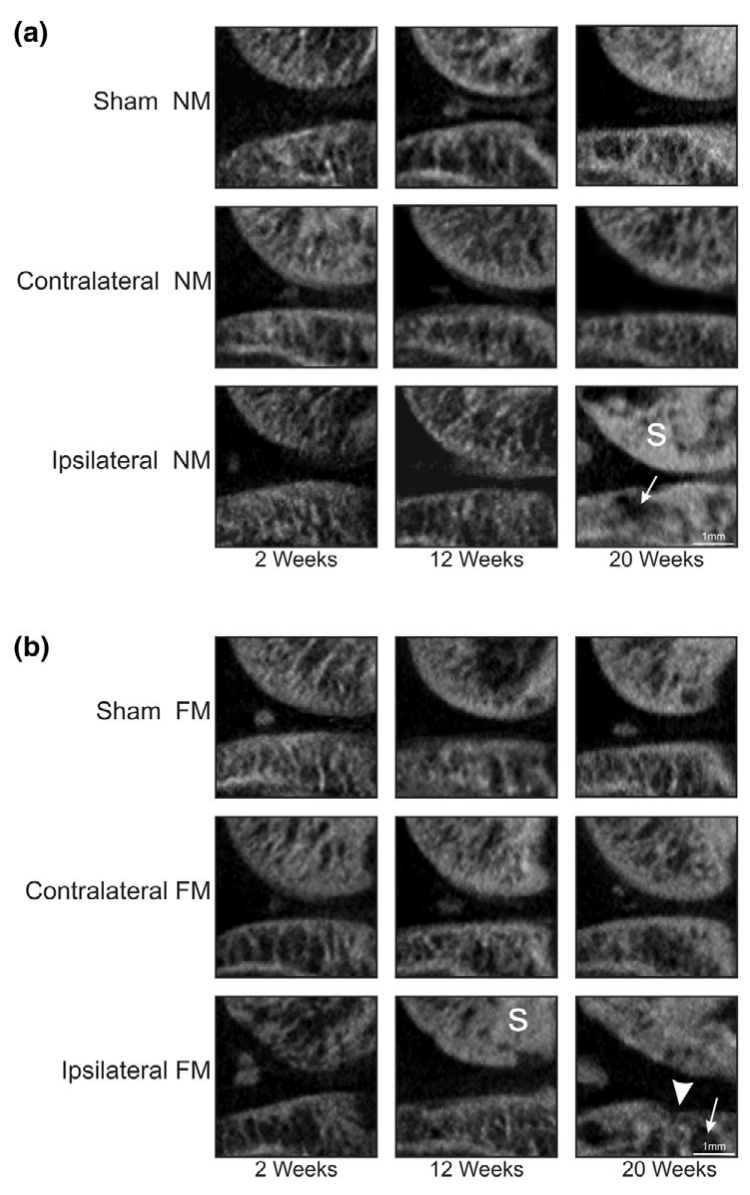
Micro-CT analysis of subchondral changes over the time course. Knee joints from **(a) **NM and **(b) **FM groups of animals were assessed by micro-CT for morphologic changes in subchondral bone in contralateral and ipsilateral joints, compared with sham controls at 2, 12, and 20 weeks. Each sagittal slice at 12 and 20 weeks is shown at the same distance into the medial joint compartment (from the medial margin) as the corresponding histologic section in Figure 2. Subchondral trabecular architecture was maintained in both sham and contralateral joints, regardless of mobilization group. Note the more extensive subchondral spaces in FM compared with NM ipsilateral joints (white arrows). Sclerotic bone (S) appeared earlier in FM (12 weeks) than in NM ipsilateral joints (20 weeks). Collapse of the subchondral plate was evident in 20 week FM joints (arrowhead). All images are shown at the same magnification, indicated by the scale bars. CT, computed tomography; FM, forced mobilization; NM, nonmobilized.

**Figure 6:**
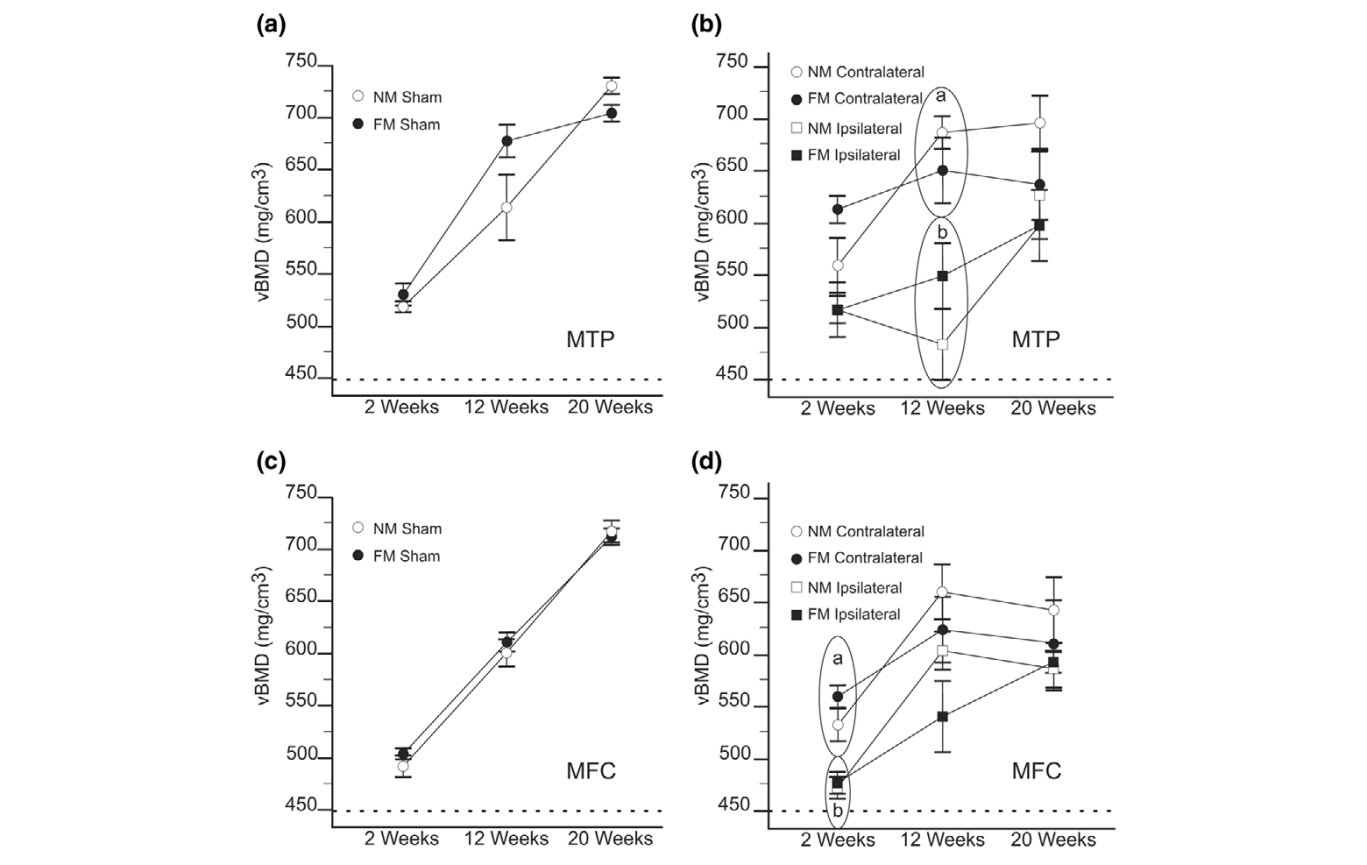
Volumetric bone mineral density analysis over the time course. Micro-CT scans were used to assess vBMD over the 20-week time course. vBMD was compared between NM and FM groups in **(a,b) **the MTP and **(c,d) **MFC of sham, and contralateral and ipsilateral treatments. Mean vBMD values ± standard error are shown. No significant effect of FM on vBMD was observed in sham, contralateral, or ipsilateral treatments (compared with NM counterparts). Contralateral vBMD means were not significantly different from sham vBMD means at any time point (statistics not indicated). Statistical analysis is done for each individual time point to indicate significantly different means among the four contralateral and ipsilateral treatments. Only at the time points where significantly different means were identified are similar means encircled, whereas significantly different means are indicated by different circles labeled a or b (*P *< 0.05; *n *= 4). CT, computed tomography; FM, forced mobilization; MFC, medial femoral compartment; MTP, medial tibial plateau; NM, nonmobilized; vBMD, volumetric bone mineral density.

We also investigated changes in vBMD and subchondral bone morphology in our model. Micro-CT analysis demonstrated that sham and contralateral joints maintained normal subchondral trabecular architecture throughout the duration of the study, regardless of mobilization group (Figure 5). vBMD increased in the MFC and MTP of NM and FM sham and contralateral joints over the 20 weeks (Figure 6). No significant effect of FM on sham or contralateral vBMD was observed (Figure 6).

However, subchondral bone of the ipsilateral joints demonstrated dramatic changes. Morphologic evaluation showed that FM ipsilateral joints developed more severe subchondral spaces (sometimes referred to as 'cysts') by 20 weeks, and loss of subchondral trabecular architecture due to sclerosis occurred earlier in FM joints (12 weeks) than in NM joints (20 weeks; Figure 5). Subchondral plate failure was also more severe in FM joints (Figure 5b). Because the micro-CT slices at 20 weeks were aligned with the corresponding histologic sections, FM ipsilateral joint subchondral plate failure can also be seen in histologic sections at 20 weeks (Figure 2b).

Interestingly, although FM affected morphology, it had no significant effect on the vBMD of the MTP and MFC of ipsilateral joints (Figure 6). However, the vBMD profiles of the ipsilateral MFC and MTP were slightly different. Whereas the vBMD of the ipsilateral MFC was reduced at 2 weeks compared with contralateral and sham MFCs (Figure 6d), the vBMD of the ipsilateral MTP was not reduced until 12 weeks (Figure 6b). By the end of the 20-week time course, MTP and MFC vBMD had recovered to sham levels, despite architectural changes. Although these results indicate that FM modifies subchondral bone morphology differently than does NM, subchondral vBMD is not further affected by FM.

**Figure 7:**
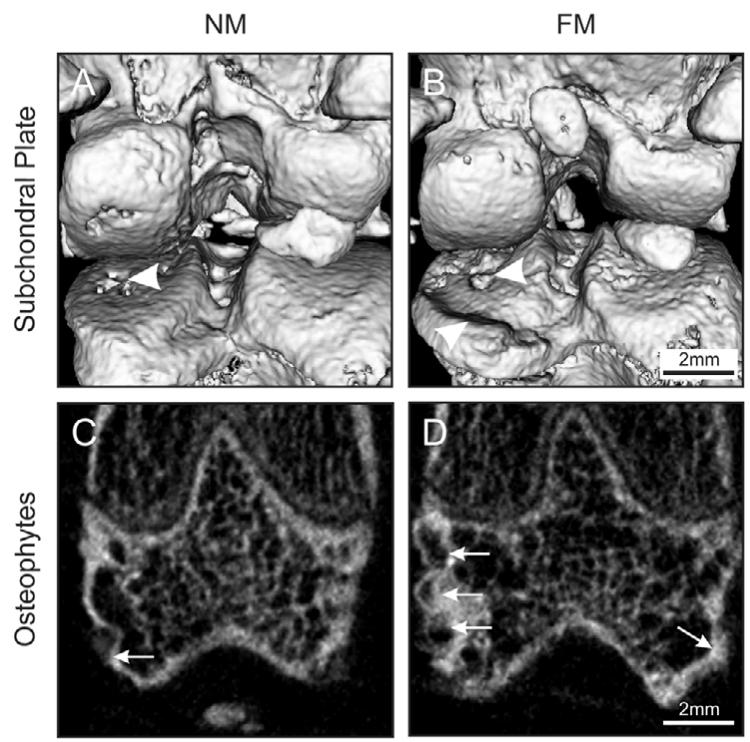
Reconstruction of micro-CT volumes reveals subchondral plate degeneration and osteophytes. Qualitative assessment of **(a,b) **subchondral plate integrity and **(c,d) **femoral osteophyte formation is shown. Reconstruction of the three-dimensional micro-CT volumes and surface rendering was used to assess the integrity of the subchondral plate in **(a) **NM and **(b) **FM ipsilateral joints at 20 weeks. Dorsal views of the reconstructed knee joints are shown. In panel a the tibial subchondral plate of NM joints exhibited minor plate breakdown (arrowhead) in the medial plateau, whereas in panel b FM plates were completely compromised by erosion and pitting (arrowheads). Coronal sections of **(c) **NM and **(d) **FM ipsilateral joints at 20 weeks reveal the presence of osteophytes (arrows). FM joints exhibit many well developed osteophytes on both medial (left) and lateral (right) joint margins, whereas NM joints show only slight medial osteophyte development (containing little mineral content). The magnification of each image is the same, indicated by the scale bars. CT, computed tomography; FM, forced mobilization; NM, nonmobilized.

Qualitative assessment of the three-dimensional micro-CT scans confirmed that NM and FM contralateral subchondral trabecular architecture, subchondral plates, and joint margins were similar to sham controls (three-dimensional analysis not shown). In the coronal and sagittal planes of NM ipsilateral joints, however, severe bone loss was evident in large spaces beneath the subchondral plates. Nonetheless, NM ipsilateral subchondral plates largely remained intact at 20 weeks. In contrast, FM ipsilateral subchondral plates deteriorated dramatically, and trabecular bone was almost completely disintegrated by 20 weeks. To demonstrate this, three-dimensional surface rendering of the subchondral plate indicated that FM joint surface topography was considerably heaved and sunken (Figure 7b), whereas NM joint surfaces remained relatively even (Figure 7a). FM also caused striking changes at the joint margins. Osteophytes were prominent along medial and lateral joint margins of FM ipsilateral joints after 20 weeks (Figure 7d), whereas very little osteophyte development was evident in NM joints (Figure 7c). Overall, FM caused more severe degradation, and stimulated the formation of features consistent with those observed in human OA (for instance, osteophytes and subchondral spaces).

### Assessment of type II collagen breakdown

**Figure 8:**
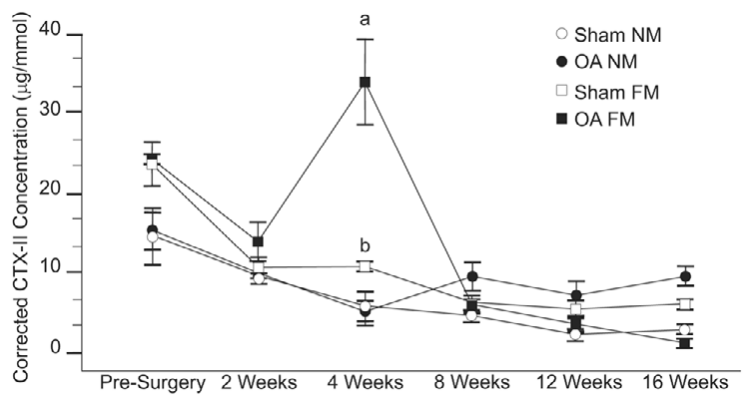
Biochemical analysis of CTX II levels as an indicator of cartilage turnover. Quantitative biochemical analysis of cartilage breakdown (type II collagen fragments) was performed on urine samples using the CTX II Pre-Clinical CartiLaps^® ^enzyme-linked immunosorbent assay. Ten animals underwent sham surgery (control) and 10 underwent OA surgery. Five animals from each group were randomly selected for FM studies, and the remaining five for NM studies. Spot urine was collected presurgically and at 2, 4, 8, 12, and 16 weeks after surgery. Samples were assayed for CTX II concentration and normalized to urine creatinine concentration. Mean CTX II concentrations corrected to creatinine ± standard error are shown. Statistical analysis was performed to test for significant differences between groups, at each time point. Only significantly different means are indicated by 'a' and 'b' (*P *< 0.05; *n *= 5). FM, forced mobilization; NM, nonmobilized; OA, osteoarthritis.

Type II collagen is the major structural component of articular cartilage [58]. When type II collagen is broken down, collagen fragments (CTX II) are released into the circulation and excreted in urine [59]. Accordingly, greater rates of cartilage breakdown cause higher levels of CTX II in urine. We investigated urine CTX II levels in this model to determine which stage(s) exhibited increased cartilage catabolism, and whether FM affected this rate. Overall, creatinine-corrected CTX II levels decreased in all sham and operated (OA) animals, with and without FM exercise, over time (Figure 8). This was most likely due to slowing growth rates of the animals (resulting in slower matrix turnover) over time. In contrast, a dramatic increase in CTX II levels occurred in FM OA animals at 4 weeks. This coincided with both the early increase in articular cartilage degradation in the histologic samples and the significantly higher OARSI scores at 4 weeks in FM ipsilateral joints. Moreover, the use of FM in OA animals increased type II collagen breakdown at earlier stages. The effects of FM on type II collagen turnover, together with the histologic findings, highlight the involvement of cartilage matrix breakdown during the early stages of OA development.

### Chondrocyte hypertrophy in cartilage degradation

**Figure 9:**
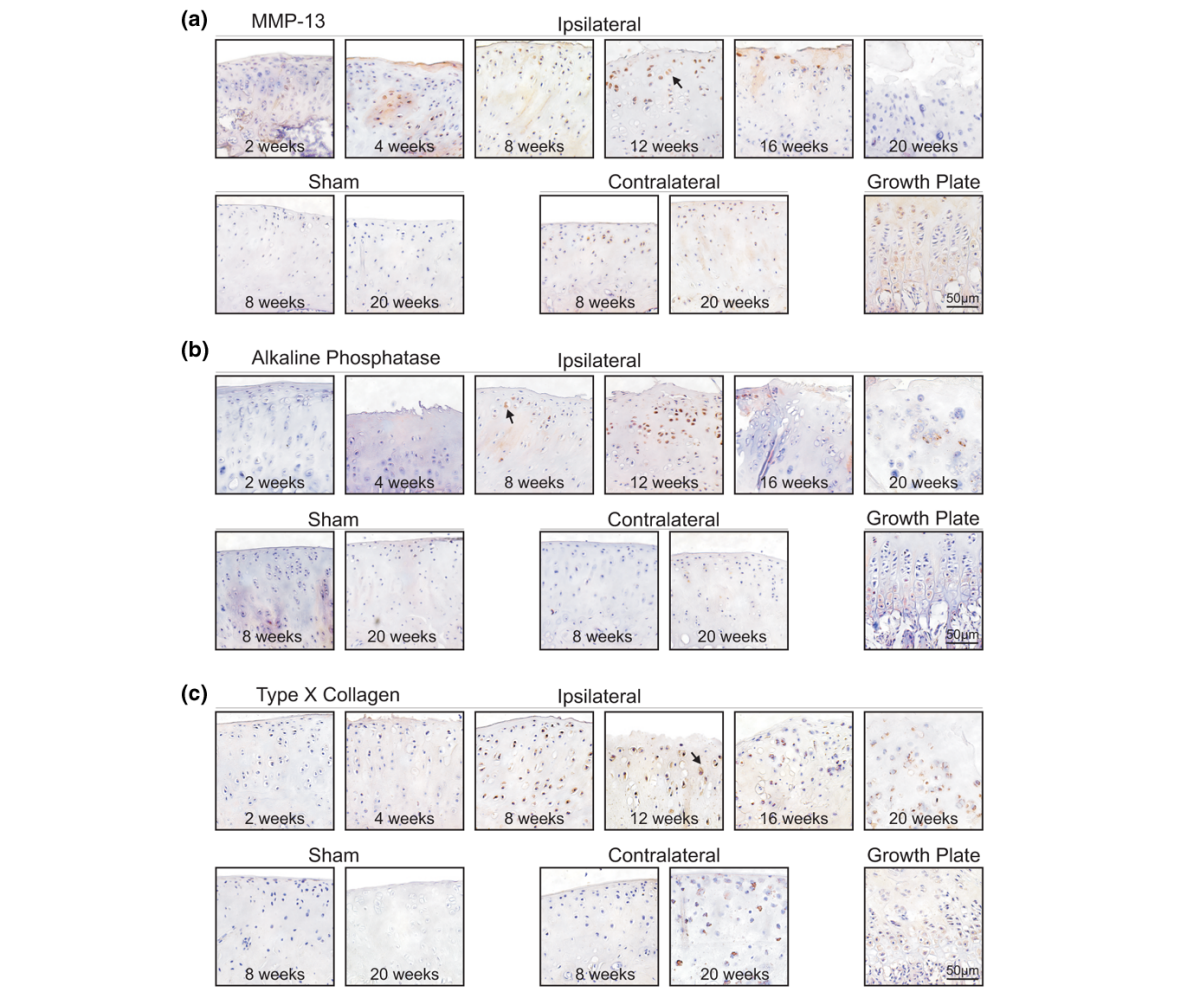
Immunostaining for markers of chondrocyte hypertrophy. Immunostaining for markers of chondrocyte hypertrophy was performed in articular cartilage sections from FM animals. Sections were probed with **(a) **anti-MMP-13, **(b) **anti-alkaline phosphatase, or **(c) **anti-type X collagen primary antibodies, followed by secondary antibodies conjugated to horseradish peroxidase. Colourimetric detection of each protein (brown precipitate) was carried out for equal time periods for all sections probed with the same primary antibody. Nuclei are counterstained with hematoxylin (blue). In the top row of each panel, representative sections from all time points following surgery in ipsilateral knee joints are shown. In the bottom row of each panel, representative sections from sham and contralateral knee joints at 8 and 20 weeks after surgery are shown. As a positive control, sections containing the growth plate are shown in each panel to demonstrate the expression of each protein in hypertrophic chondrocytes. Articular chondrocytes with a hypertrophic-like morphology are also indicated (arrows). Experiments for each protein were carried out on sections from at least three different animals with reproducible results. All images are shown at the same magnification, as indicated by the scale bars. FM, forced mobilization; MMP, matrix metalloproteinase.

Because our histologic analyses of FM ipsilateral articular cartilage revealed the presence of mid-zone chondrocytes with a hypertrophic appearance, we assessed the expression of several hypertrophic marker proteins in the articular cartilage of FM animals. Immunohistochemistry was used to assess the spatial and temporal expression of MMP-13, alkaline phosphatase, and type X collagen over time (Figure 9). These proteins were not expressed at any time point in FM sham articular cartilage. MMP-13 expression was observed at 4 weeks in ipsilateral samples, and was expressed earlier than alkaline phosphatase and type X collagen (Figure 9a). MMP-13 continued to be expressed at high levels (compared with sham) until 20 weeks. Some MMP-13 staining was also observed in contralateral cartilage. Alkaline phosphatase expression was increased in ipsilateral chondrocytes by 8 weeks, increased dramatically at 12 weeks, and diminished thereafter (Figure 9b). No alkaline phosphatase staining was observed in contralateral samples. Type X collagen was expressed in ipsilateral cartilage as early as 8 weeks and continued to be expressed through to 20 weeks (Figure 9c). Contralateral samples also exhibited type X collagen staining but only at 20 weeks. Growth plate analysis confirmed expression of all three proteins in hypertrophic chondrocytes and was used as a positive control for each marker. In addition, the morphologic appearance of chondrocytes in ipsilateral cartilage suggested larger cells, with larger lacunae, than that of chondrocytes in sham cartilage. These results indicate that articular chondrocytes in FM cartilage undergo hypertrophic-like differentiation. Contralateral chondrocytes also appeared to be affected, albeit to a lesser extent.

## Discussion

Development of preclinical OA models is crucial to the study of OA pathophysiology and evaluation of DMOAD efficacy. However, models must be extensively characterized to ensure that appropriate conclusions are drawn from the studies that use them. In this study we characterized a surgical rodent model of OA, in which ACL-T and PM lead to joint destabilization, and thus OA pathology. Notably, this model more closely reflects secondary forms of OA, which arise from trauma or other disorders [60]. Nonetheless, it may also have application in primary OA studies. We evaluated OA activity through histomorphometric analysis using the quantitative OARSI scoring method [42], quantitative analysis of bone mineral density [61,62], and biochemical analysis of cartilage breakdown [40,41]. Furthermore, we are the first to evaluate the effects of FM on pathogenesis in a rat model of OA, and we assessed chondrocyte hypertrophy in OA pathogenesis. To date, a comprehensive, longitudinal evaluation of a preclinical surgical rodent model of OA, as shown here, has not been reported.

Our histologic results indicate that in this model, articular cartilage degradation consistently begins as early as 2 weeks after surgery and is worse with FM. Early in pathogenesis, the profile of cartilage degradation initially reflects the edema and delamination of the superficial layer, and development of fissures into the mid-zone that are commonly observed during early stages of human OA [60]. At later time points the model also exhibits features characteristics of late-stage human OA including denudation, and osteophytes and fibrocartilage-like tissue are present at the denuded surface when FM is applied [60,63]. Interestingly, although proteoglycan loss occurred at earlier stages in regions with more severe lesions, proteoglycan loss was not progressive over the time course. In fact, proteoglycan staining was more intense near the end of the time course, particularly in repair tissues, which is probably due to a compensatory anabolic repair response. Accordingly, quantitative analyses that include proteoglycan loss in addition to other features of degradation are necessary to achieve a comprehensive understanding of disease progression.

In addition, early loss of subchondral bone density and trabecular architecture were also present and are reminiscent of human OA [10]. Ultimately, these properties are likely to persist to end-stage OA, where joint failure occurs and invasive arthroplastic intervention is required. Longitudinal analysis allows evaluation of both the early and late stages of OA development. This is highly effective in rodent models in particular, because the time course to overt pathology is relatively short, and a larger number of animals can be managed. As previously shown, longitudinal three-dimensional vBMD analysis in rabbits [52] and OARSI scores in rodents [42] are precise tools for assessing OA development in animal models. Overall, our findings correspond with current assessments of OA in humans, and the model produces significant, predictable, and reproducible results. Therefore, we conclude that the ACL-T/PM model of OA with FM is appropriate for use in preclinical studies of OA, providing a means to study the onset, development, and characteristics of lesions that appear to be similar to those in human OA. However, although this model mimics certain aspects of human OA, extrapolation of joint lesions to human OA should be considered with caution, as with any animal model.

There is often disagreement as to whether sham surgery is the most appropriate control in surgical models of OA [28,64]. Accordingly, in addition to the ipsilateral knee, we investigated OA activity in the contralateral joint. Histology and OARSI scores revealed minor OA activity in contralateral articular cartilage, including significantly higher scores in contralateral joints at 2 and 12 weeks, compared with shams. We also saw induction of type X collagen and MMP-13 expression in contralateral cartilage late in the 5-month time course. Evidence of contralateral OA activity, however, did not worsen over the time course. Nonetheless, these findings indicate that the surgically unaltered contralateral joint is affected to a minor extent by OA induction in the model. The effects may be due to alterations in weight-bearing during rest or activity [65] or to systemic factors (for example, circulating inflammatory factors [66,67]) yet to be identified in this model [68]. Interestingly, subchondral bone and vBMD profiles were not altered in contralateral joints (compared with sham joints), perhaps protecting them from OA advancement. Furthermore, we recently demonstrated that contralateral chondrocyte gene expression profiles are altered, relative to shams, emphasizing the importance of sham controls in gene expression studies [69]. By extension, the contralateral joint may be susceptible to developing OA caused by changes in gait or systemic effects. Accordingly, we conclude that a sham operation in independent animals is the most appropriate control in genetic and biochemical studies [70,71]. However, in studies focused on subchondral bone (for example, micro-CT studies) the contralateral joint is a sufficient control, because we did not observe any contralateral changes in subchondral architecture or vBMD over 5 months (compared with shams), and is advantageous for controlling inter-animal variables such as age and weight.

It is thought that OA may arise from abnormal articulations and repetitive joint loading. Accordingly, we explored the effects of FM (inducing repetitive joint loading and maximal flexion and extension of the knee joint) on OA development in the ACL-T/PM model. Because NM animals in this study could voluntarily remain stationary, it was hypothesized that such a paradigm of FM would accelerate pathogenesis. Indeed, this was the case. FM animals had increased cartilage degradation at 2 weeks after surgery, higher OARSI scores, articular surface deformation and subchondral plate failure, and earlier subchondral bone sclerosis than did NM animals. Similar to human OA, FM also induced abnormal repair processes, including fibrocartilage-like tissue and osteophytes, which were not present in NM animals. This was probably due to subchondral plate failure caused by FM, allowing infiltration of bone marrow stromal cells and activation of repair processes such as that seen following osteochondral fracture [72]. Furthermore, biochemical analysis of type II collagen breakdown indicated that cartilage degradation is elevated in FM animals at 4 weeks. Together, these data indicate that FM in this model effectively accelerates both the onset and progression of OA pathogenesis, resulting in a disease state that is reminiscent of human OA.

The clinical implications of FM are unclear. FM exercise, as applied in this study, involves walking slowly on a rotating drum for 30 min, three times per week, and thus is not vigorous exercise. Rather, FM forces maximal joint flexion and extension and causes repetitive increased load bearing that is not necessarily exhaustive but is deleterious when applied to the destabilized joint. Importantly, however, FM exercise had no deleterious effects on nondestabilized joints. Although we know from this and many other previous studies that joint destabilization in animal models leads to OA pathogenesis, we conclude here that FM accelerates OA pathogenesis, and that repetitive load bearing exercise is deleterious to the destabilized joint, at least in this OA model. A question faced by many patients who suffer OA is whether they should exercise. Exercise is often recommended for patients with hip or knee OA [73], and the American College of Rheumatology recommends aerobic exercise for patients with knee and hip OA [74]. However, fear that exercise is damaging their joints causes some patients with OA not to exercise [75]. The therapeutic importance of exercise is therefore a complex issue. One point of departure may be in the form of exercise. Indeed, many studies have demonstrated the beneficial effects of light to moderate exercise in animal models of OA and patients [35,76-78], whereas more strenuous exercise and repetitive load bearing has a deleterious effect, at least in experimental animals [39]. We must stress that our study does not suggest that nonrepetitive load bearing forms of exercise accelerate pathogenesis, nor that they are detrimental. However, our study does indicate that both the type and timing of exercise after a joint injury should be considered with care, in addition to the stability of the joint in question.

Several studies have proposed that recapitulation of hypertrophic chondrocyte differentiation is involved in cartilage degradation in OA [79,80]. We investigated this hypothesis in our OA model by analyzing the expression of hypertrophic chondrocyte markers, including MMP-13, alkaline phosphatase, and type X collagen. We demonstrated that all three markers of chondrocyte hypertrophy are increased in FM ipsilateral cartilage, supporting this hypothesis. MMP-13, when activated, will digest the territorial matrix, and deposition of type X collagen and secretion of factors that facilitate calcification of the cartilage matrix (such as alkaline phosphatase) are deleterious to articular cartilage. Furthermore, degradation of the matrix by catabolic factors such as MMP-13 make the cartilage susceptible to further mechanical erosion. Accordingly, hypertrophic differentiation of articular chondrocytes will facilitate degradation in OA. Moreover, we conclude that hypertrophic-like differentiation of articular chondrocytes is one facet of, and probably contributes to, OA pathology in this model.

Preclinical studies are often limited by time or financial constraints. Consequently, accelerating pathology with FM (as presented here), while maintaining similarity to human OA, will maximize productivity in preclinical studies. Of course, no OA model can reproduce human OA pathology identically [81]. For example, the NM animals used in this study did not develop osteophytes, which are known to develop in human knee OA [82,83]. Interestingly, the use of FM induced osteophyte formation. Such limitations must be considered when designing and drawing conclusions from a preclinical OA model. Instead, OA models are highly useful for investigating specific properties of OA. For example, this model accurately mimics the articular cartilage degradation and subchondral changes that are observed in many types of human OA, such as post-traumatic arthritis [84]. Although we were unable to monitor disease progression in individual animals over time (because of sacrifice for histology), we now have a predictable index of pathogenesis in this model. We propose that future studies on disease progression in the joint should use the 2-week to 8-week time points for early, and 12-week to 20-week time points for late stages of OA, and are most relevant to human OA when combined with FM in this model. Thus, future studies will require only end-point histology, and disease progression may be monitored solely with *in vivo *techniques such as high resolution magnetic resonance imaging [85,86] and micro-CT.

## Conclusion

Development and comprehensive characterization of pre-clinical OA models is pivotal to understanding pathology and intervention mechanisms. Here, we report a comprehensive, longitudinal characterization of the ACL-T/PM rat model of OA, using multiple methodologies and measurement tools. We found that articular cartilage degradation, subchondral deformation, and biochemical urinalysis profiles correlate with current understanding of OA pathology. Furthermore, we demonstrated that FM is useful for accelerating OA onset and severity. This model is not meant to replace other available models, but it highlights the advantages of small animal studies and the variety of experimental techniques that may be used in such investigations. For example, we recently completed a parallel study in which we evaluated genome-wide changes in chondrocyte gene expression in degrading articular cartilage; this preclinical study would not have been possible in larger animals (which would be less relevant to humans from a genetic standpoint) with currently available gene expression tools [69]. We propose that the ACL-T/PM model is suitable for preclinical studies of OA, including studies investigating the efficacy of DMOADs.

## Abbreviations

ACL-T = anterior cruciate ligament transection; CT = computed tomography; DMOAD = disease-modifying osteoarthritis drug; FM = forced mobilized; MFC = medial femoral compartment; MMP = matrix metalloproteinase; MTP = medial tibial plateau; NM = nonmobilized; OA = osteoarthritis; OARSI = Osteoarthritis Research Society International; PM = partial medial meniscectomy; ROI = region of interest; vBMD = volumetric bone mineral density.

## Competing interests

The authors declare that they have no competing interests.

## Authors' contributions

CTGA participated in the design of the study, carried out all histologic, biochemical, and immunoassays, performed statistical analyses, and drafted the manuscript. Coauthor DDM carried out micro-CT data acquisition and analysis, and performed statistical analysis. VP and NS were involved in development of the OA model and editing of the manuscript. SMB, JLH, and DWH were involved in the development of the model, design of the study, and editing the manuscript. FB participated in the conception, design, and coordination of the study, and helped to draft the manuscript. All authors read and approved the final manuscript.

## Figures and Tables

**Table 1:** Summary of surgical and mobilization treatment groups

Surgical treatment group	Number of animals per time point
Nonmobilization groups	
Sham	4
Contralateral	4
Ipsilateral	4
Forced mobilization	
Sham	4
Contralateral	4
Ipsilateral	4
